# Comparison of low-contrast detectability between uniform and anatomically realistic phantoms—influences on CT image quality assessment

**DOI:** 10.1007/s00330-021-08248-3

**Published:** 2021-09-02

**Authors:** Juliane Conzelmann, Ulrich Genske, Arthur Emig, Michael Scheel, Bernd Hamm, Paul Jahnke

**Affiliations:** 1 Department of Radiology, Charité – Universitätsmedizin Berlin, corporate member of Freie Universität Berlin, Humboldt-Universität zu Berlin, and Berlin Institute of Health, Charitéplatz 1, 10117 Berlin, Germany; 2 Data Analytics and Computational Statistics, Hasso Plattner Institute, Digital Engineering Faculty, University of Potsdam, 14482 Potsdam, Germany; 3 Department of Neuroradiology, Charité – Universitätsmedizin Berlin, corporate member of Freie Universität Berlin, Humboldt-Universität zu Berlin, and Berlin Institute of Health, Charitéplatz 1, 10117 Berlin, Germany; 4 Berlin Institute of Health (BIH), Anna-Louisa-Karsch-Str. 2, 10178 Berlin, Germany

**Keywords:** Tomography, X-ray computed, Phantoms, imaging, Medical physics, Neck, Radiation protection

## Abstract

**Objectives:**

To evaluate the effects of anatomical phantom structure on task-based image quality assessment compared with a uniform phantom background.

**Methods:**

Two neck phantom types of identical shape were investigated: a uniform type containing 10-mm lesions with 4, 9, 18, 30, and 38 HU contrast to the surrounding area and an anatomically realistic type containing lesions of the same size and location with 10, 18, 30, and 38 HU contrast. Phantom images were acquired at two dose levels (CTDIvol of 1.4 and 5.6 mGy) and reconstructed using filtered back projection (FBP) and adaptive iterative dose reduction 3D (AIDR 3D). Detection accuracy was evaluated by seven radiologists in a 4-alternative forced choice experiment.

**Results:**

Anatomical phantom structure impaired lesion detection at all lesion contrasts (*p* < 0.01). Detectability in the anatomical phantom at 30 HU contrast was similar to 9 HU contrast in uniform images (91.1% vs. 89.5%). Detection accuracy decreased from 83.6% at 5.6 mGy to 55.4% at 1.4 mGy in uniform FBP images (*p* < 0.001), whereas AIDR 3D preserved detectability at 1.4 mGy (80.7% vs. 85% at 5.6 mGy, *p* = 0.375) and was superior to FBP (*p* < 0.001). In the assessment of anatomical images, superiority of AIDR 3D was not confirmed and dose reduction moderately affected detectability (74.6% vs. 68.2%, *p* = 0.027 for FBP and 81.1% vs. 73%, *p* = 0.018 for AIDR 3D).

**Conclusions:**

A lesion contrast increase from 9 to 30 HU is necessary for similar detectability in anatomical and uniform neck phantom images. Anatomical phantom structure influences task-based assessment of iterative reconstruction and dose effects.

**Key Points:**

• A lesion contrast increase from 9 to 30 HU is necessary for similar low-contrast detectability in anatomical and uniform neck phantom images.

• Phantom background structure influences task-based assessment of iterative reconstruction and dose effects.

• Transferability of CT assessment to clinical imaging can be expected to improve as the realism of the test environment increases.

**Supplementary Information:**

The online version contains supplementary material available at 10.1007/s00330-021-08248-3.

## Introduction

Image properties of clinical computed tomography (CT) images vary significantly due to differences between vendors, scanner generations, software versions, imaging techniques, and reconstruction methods. This diversity affects the diagnostic quality of CT images [1], and differences are likely to increase further as CT techniques evolve. In light of this situation, it is of relevance to ensure objective assessment and comparison of the clinical performance of CT techniques [2]. Task-based methods have been proposed for that purpose and should be applicable to evaluate the diagnostic performance of CT images regardless of the underlying imaging technology used [3, 4].

Task-based assessment is typically used to test lesion detectability in CT images of uniform phantoms, and it is commonly assumed that the results can be transferred to CT images of patients acquired in the clinical setting. Yet, there is evidence that uniform phantoms may not reflect clinical performance adequately. First, previous X-ray studies have shown that background structure affects detectability and conclusions about dose effects on image quality [5–7]. Second, background texture has also been identified to affect detectability and estimated dose reduction potential of an iterative reconstruction algorithm in a CT study [8]. Conversely, the authors of another CT study report only negligible texture effects, concluding that uniform phantoms may allow sufficient assessment of clinical performance [9]. Both of these CT studies investigated cropped images mimicking vessel-free liver textures. In order to better understand the validity of CT assessment with uniform phantoms for clinical imaging, it would be desirable to evaluate how such assessments relate to CT images obtained in phantoms with full anatomical detail.

A recent study introduced anatomically realistic neck phantoms that can be used for such purposes [10]. The phantoms investigated in that study contained low-contrast lesions and were produced using radiopaque 3D printing based on a neck CT image of a patient. Another recent study used the same CT image as a template to produce a uniform neck phantom for low-contrast detectability experiments [11]. The present study compares low-contrast detectability between these two types of phantoms to test the hypothesis that anatomical detail affects task-based CT assessment. CT images of the phantoms acquired at two dose levels and reconstructed with filtered back projection and an iterative reconstruction algorithm were analyzed. The overall aim was to evaluate the effects of anatomical background structure on task-based image quality assessment in comparison with a uniform phantom background.

## Methods

### Study design

Neck-shaped phantoms with uniform and anatomical texture and hypodense lesions of 10 mm diameter and 4 to 38 HU contrast were imaged with two dose levels. Images were reconstructed with filtered back projection (FBP) and adaptive iterative dose reduction 3D (AIDR 3D). Lesion detectability was assessed by seven radiologists and compared between background types, dose levels, and reconstruction methods.

### Phantoms

**Fig. 1:**
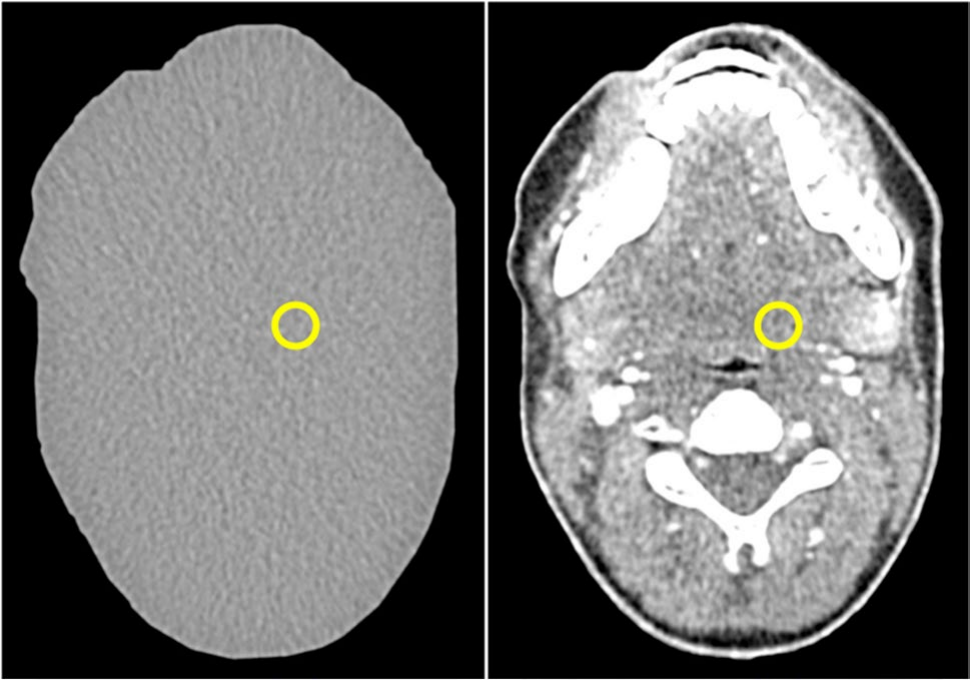
CT images of the uniform and the anatomical phantom. The yellow circle indicates the lesion position

Two phantom types, which were previously introduced for low-contrast detectability experiments, were used for this study: a uniform type consisting of polymethyl methacrylate with the shape of a patient’s neck and a 3D printed, anatomically realistic type of identical shape [10, 11]. All phantoms had the same dimension of 15.4 cm (length) × 10.6 cm (width). Six different versions of the uniform phantom type and five versions of the anatomical type were used. One version of each type did not contain any lesion. The other versions each contained a single low-contrast lesion of 10 mm diameter in the left parapharyngeal space. The lesion was in the same position in all phantoms. Lesion contrasts were 4, 9, 18, 30, and 38 HU (uniform phantom) and 10, 18, 30, and 38 HU (anatomical phantom). The lesion contrasts were validated in previous studies by HU measurement in 2700 images acquired with six different dose levels (uniform phantom) and in 2808 images acquired with twenty-seven different dose levels (anatomical phantom) [10, 11]. In these validation experiments, lesion contrast was calculated as HU difference between regions of interest (ROIs) of 0.5 cm^2^ inside the lesions and six ROIs of 4.9 cm^2^ (uniform phantom) and one ROI of 3 cm^2^ (anatomical phantom) surrounding the lesions. The lesions were rod-shaped, and the phantoms were constructed in such a way that multiple adjacent images displaying the same lesion and phantom background could be extracted per CT acquisition. Figure 1 shows a CT image of each phantom type and indicates the lesion position. Details on phantom construction, acquisitions, and measurements performed for evaluating lesion contrasts can be found elsewhere [10, 11].

### Image acquisition

CT images of the uniform phantom originated from a previous study [11] and were acquired on a Canon Aquilion Prime CT scanner (Canon Medical Systems). CT images of the anatomical phantoms were acquired on the same system using identical parameters: helical mode, tube voltage of 120 kVp, fixed collimation of 80 × 0.5 mm, rotation time of 0.5 s, 0.813 pitch, and a 280 mm diameter field of view. A 30- and 120-mA tube currents were used, corresponding to CTDIvol values of 1.4 and 5.6 mGy. Five acquisitions were performed per tube current. Images were reconstructed with 0.5-mm slice thickness and a soft tissue kernel (FC08) using FBP and AIDR 3D. For the subsequent detectability experiment, four CT images were extracted per acquisition of the lesion-bearing phantoms with 9, 18, 30, and 38 HU lesion contrast (uniform phantom) and 10, 18, 30, and 38 HU lesion contrast (anatomical phantom). Thus, a total of 640 lesion-bearing images were extracted (2 phantom types × 4 lesion contrasts × 2 tube currents × 2 reconstruction methods × 5 repeated acquisitions × 4 images).

### Detectability experiment

Each lesion-bearing image was paired with three non-lesion-bearing images of the corresponding phantom type (uniform or anatomical), which were acquired and reconstructed with identical parameters. Each of the resulting 640 image quartets was presented to seven radiologists in a 4-alternative forced choice (4-AFC) experiment. Readers were asked to select the image containing a lesion and to indicate their confidence using a five-step scale ranging from 1 = not confident to 5 = confident. Readings were performed using in-house developed software on diagnostic screens (Eizo RadiForce RX250, Eizo Corporation). In addition to the reading results obtained here, results from a previous reading experiment performed with images of the uniform phantom and 4 HU lesion contrast were included in the analysis [11]. Image acquisitions and readings in that previous study were performed in the same way as in the present study (i.e., the same CT system, acquisition and reconstruction parameters, 4-AFC methodology, and readers were involved). The results were included to complement the current data used to analyze dose and image reconstruction effects in uniform phantoms.

### Noise characteristics

The standard deviation (SD) of pixel values and the noise power spectrum (NPS) were measured using 200 images per phantom type, tube current, and reconstruction method. In each image, a square ROI of 32 × 32 pixels (17.5 × 17.5 mm) was placed in the same location in the parapharyngeal space adjacent to the lesion. The ROI position was selected to include a fairly homogeneous area of the anatomical phantoms. A larger ROI size or multiple ROIs would have led to the inclusion of largely inhomogeneous areas of the anatomical phantoms such as the mandibula or vascular structures. Also, ROI placement inside the lesions was not possible because the lesion size was too small to perform NPS measurement. The 2D NPS was calculated using the following Eq. (1):

1 $$\mathrm{NPS}\left({f}_{x},{f}_{y}\right)= \frac{{b}_{x}{b}_{y}}{{L}_{x}{L}_{y}} {\langle {\left|{\mathrm{FFT}}_{2D}\left[\mathrm{ROI}\left(x,y\right)- {\mathrm{ROI}}_{\mathrm{Background}}\left(x,y\right)\right]\right|}^{2}\rangle }_{{N}_{\mathrm{ROI}}}$$

where *b*_x_ and *b*_y_ are the pixel sizes (0.546 mm) in the x- and y-direction, respectively, and *L*_x_ and *L*_y_ are the ROI lengths (17.5 mm) in the x- and y-direction, respectively. FFT_2D_ is the 2D fast Fourier transform. ROI_Background_ is the background noise in ROI(*x*,*y*) measured using second-order polynomial fitting by minimizing the residual sum of squares [12]. *N*_ROI_ is the number of ROIs (200) per phantom type, tube current, and image reconstruction that was used to average the squared amplitude of the fast Fourier transform.

### Data and statistical analysis

Detection accuracy was calculated as the percentage of correct lesion image selections per reader. Detection accuracy and reader confidence were compared between uniform and anatomical phantom backgrounds using t-tests. Results were compared between dose levels and reconstruction methods with analysis of variance for repeated measurement using post hoc tests with Tukey’s method to adjust for multiple comparisons. Differences were interpreted as significant for *p* < 0.05.

## Results

### Comparison of phantom types

**Fig. 2:**
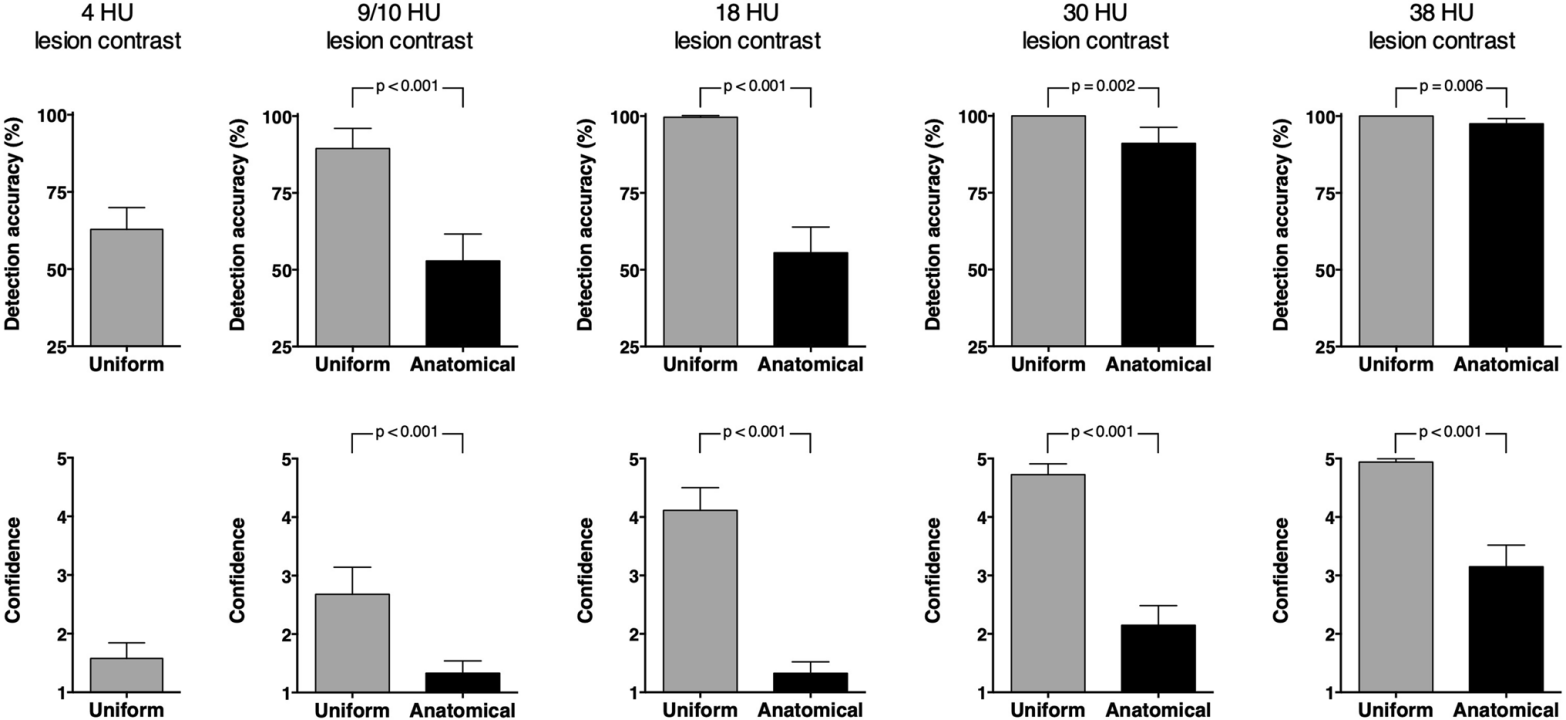
Detection accuracy and reader confidence in uniform and anatomical phantom images. Averaged results across all readers, dose levels, and reconstruction methods at 4, 9 (uniform), 10 (anatomical), 18, 30, and 38 HU lesion contrast are presented. Error bars indicate 95% confidence intervals. 25% detection accuracy corresponds to random guessing (no detection)

Figure 2 shows a comparison of detection accuracy and reader confidence between uniform and anatomical phantoms. Averaged results across all readers, dose levels, and reconstruction methods are presented. Phantom background texture significantly affected detectability at all lesion contrasts. Readings of images of the uniform phantom yielded high detection accuracy already at relatively low lesion contrast of 9 HU (89.5%, 95% CI: 82.9 to 96%), which improved to 99.6% (95% CI: 99.1 to 100.2%) at 18 HU and perfect detection at 30 and 38 HU contrast. Conversely, readings of images of the anatomical phantoms yielded low detection accuracy at 10 HU (52.9%, 95% CI: 44.1 to 61.6%) and 18 HU (55.5%, 95% CI: 47.2 to 63.9%), which improved to 91.1% (95% CI: 85.8 to 96.3%) at 30 HU and 97.5% (95% CI: 95.8 to 99.2%) at 38 HU contrast. Clear differences between uniform and anatomical images were also observed for reader confidence (Fig. 2, suppl. table 1). Similar detection accuracies for the two phantom types were achieved when comparing 9 HU lesion contrast in the uniform phantom and 30 HU contrast in the anatomical phantom (89.5% vs. 91.1%, *p* = 0.587). Readings of images of the uniform phantom with 4 HU lesion contrast originating from a previous study yielded an average detection accuracy of 62.9% across all readers, dose levels, and reconstruction methods (95% CI: 55.8 to 69.9%) [11].

### Comparison of dose and image reconstruction

**Fig. 3:**
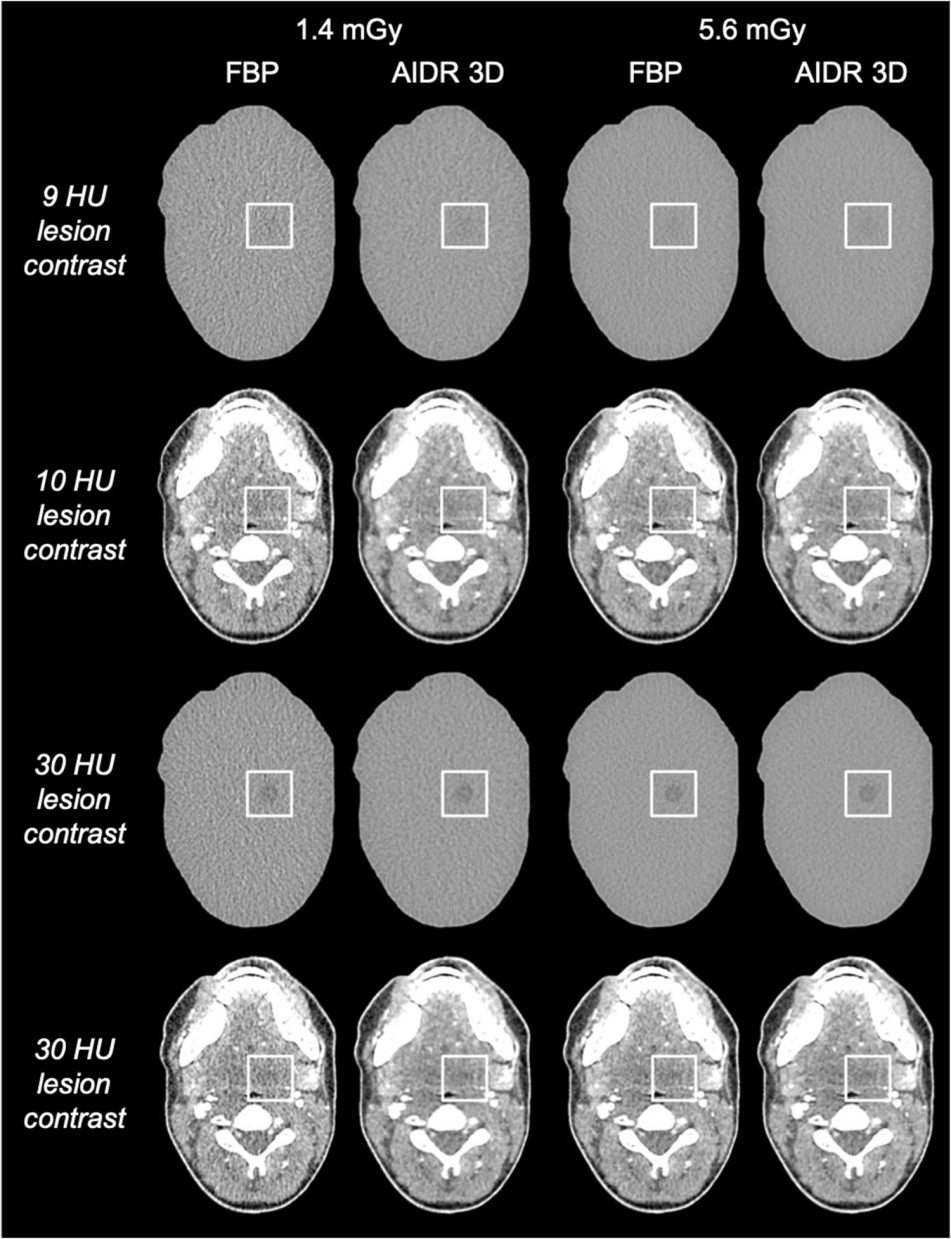
Uniform and anatomical phantom images acquired at 1.4 and 5.6 mGy and reconstructed with filtered back projection (FBP) and adaptive iterative dose reduction 3D (AIDR 3D)

Figure 3 provides a series of uniform and anatomical phantom images acquired at 1.4 and 5.6 mGy and reconstructed with FBP and AIDR 3D. The figure includes uniform images with 9 HU lesion contrast and anatomical images with 30 HU lesion contrast, which yielded similar overall detection accuracies. Detailed detection accuracy results per dose, reconstruction method, and lesion contrast are presented in Tables 1 and 2.

**Table 1:** Detection accuracy (%) per dose, reconstruction method, and lesion contrast in uniform phantom images. Means and 95% confidence intervals are presented

		4 HU lesion contrast	9 HU lesion contrast	18 HU lesion contrast	30 HU lesion contrast	38 HU lesion contrast
1.4 mGy	FBP	42.9 (27.3 to 58.4)	67.9 (48.8 to 86.9)	99.3 (97.5 to 101)	100	100
	AIDR 3D	67.9 (57.9 to 77.8)	93.6 (84.4 to 102.7)	100	100	100
5.6 mGy	FBP	70 (55.9 to 84.1)	97.1 (93.5 to 100.8)	99.3 (97.5 to 101)	100	100
	AIDR 3D	70.7 (56.7 to 84.7)	99.3 (97.5 to 101)	100	100	100

**Table 2:** Detection accuracy (%) per dose, reconstruction method, and lesion contrast in anatomical phantom images. Means and 95% confidence intervals are presented

		10 HU lesion contrast	18 HU lesion contrast	30 HU lesion contrast	38 HU lesion contrast
1.4 mGy	FBP	48.6 (26.3 to 70.9)	45 (23 to 67)	85.7 (66.2 to 105.2)	93.6 (89.2 to 98)
	AIDR 3D	47.9 (25.5 to 70.2)	54.3 (36.5 to 72.1)	90.7 (79.3 to 102.2)	99.3 (97.5 to 101)
5.6 mGy	FBP	48.6 (26.3 to 70.9)	56.4 (41.6 to 71.2)	95.7 (89 to 102.5)	97.9 (92.6 to 103.1)
	AIDR 3D	66.4 (51.4 to 81.5)	66.4 (43.2 to 89.7)	92.1 (82.2 to 102.1)	99.3 (97.5 to 101)

**Fig. 4:**
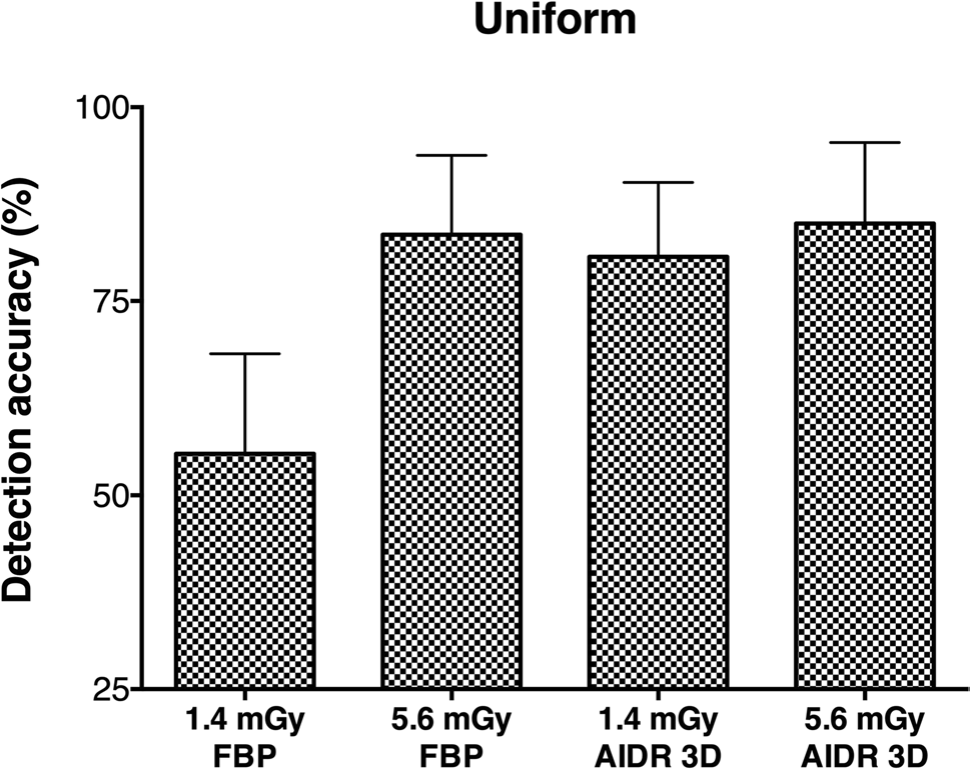
Detection accuracy per dose and reconstruction method in uniform phantom images. Averaged results across 4 and 9 HU lesion contrast are presented. Error bars indicate 95% confidence intervals. 25% detection accuracy corresponds to random guessing (no detection)

At 18 HU lesion contrast and above, readings of images of the uniform phantom reached 100% detection accuracy and could therefore not be used for the analysis of dose and image reconstruction effects. Results for 4 and 9 HU lesion contrast are summarized in Table 3 and presented in Fig. 4. Dose reduction from 5.6 to 1.4 mGy decreased lesion detectability in uniform images that were reconstructed with FBP (83.6% vs. 55.4%, *p* < 0.001). AIDR 3D maintained detectability (85% vs. 80.7%, *p* = 0.375) and was superior to FBP at 1.4 mGy (*p* < 0.001). Analysis of the uniform phantom thus showed strong dose effects on FBP-reconstructed images and superiority of AIDR 3D at 1.4 mGy.

**Table 3:** Detection accuracy (%) per dose and reconstruction method in uniform phantom images. Averaged results across 4 and 9 HU lesion contrast and 95% confidence intervals are presented

	FBP	AIDR 3D	*p* value
1.4 mGy	55.4 (42.5 to 68.2)	80.7 (71.1 to 90.3)	< *0.001*
5.6 mGy	83.6 (73.4 to 93.8)	85 (74.6 to 95.4)	*0.785*
*p* value	< *0.001*	*0.375*

**Fig. 5:**
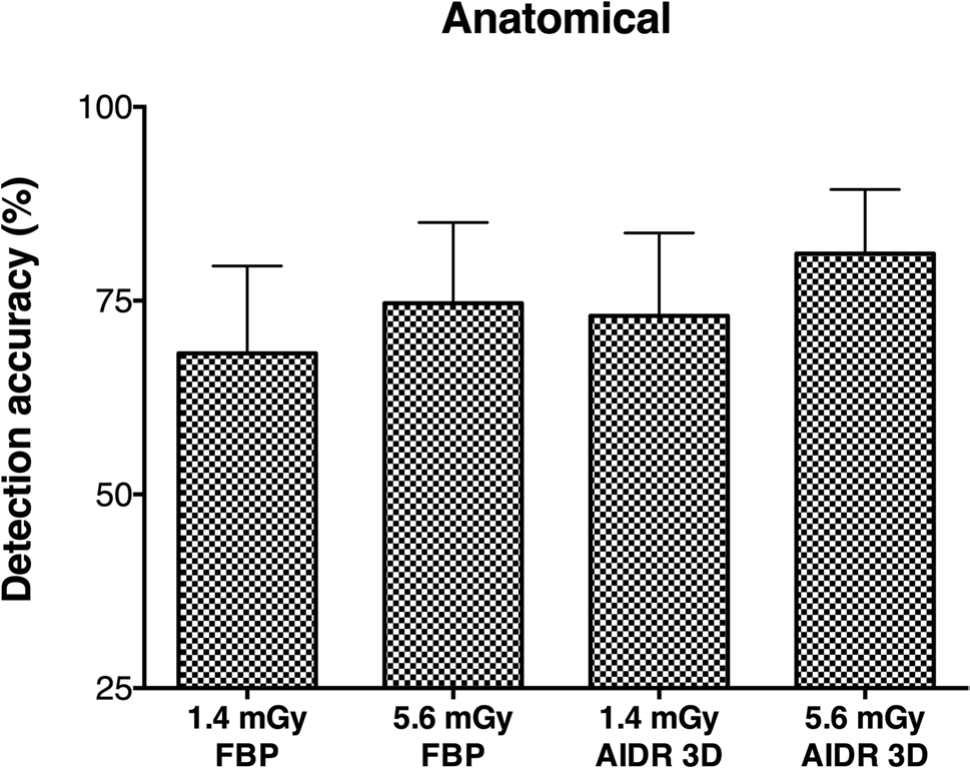
Detection accuracy per dose and reconstruction method in anatomical phantom images. Averaged results across all lesion contrasts are presented. Error bars indicate 95% confidence intervals. 25% detection accuracy corresponds to random guessing (no detection)

Figure 5 shows the effects of dose and image reconstruction on detection in anatomical phantoms. Numerical results are provided in Table 4. In contrast to the uniform phantom, AIDR 3D did not show clear advantages over FBP at any dose level (73% vs. 68.2%, *p* = 0.144 at 1.4 mGy and 81.1% vs. 74.6%, *p* = 0.111 at 5.6 mGy). Moreover, the strong effects of dose reduction on FBP-reconstructed images were not confirmed. Instead, dose reduction moderately affected detectability in a similar manner for both reconstruction methods (*p* = 0.027 for FBP and *p* = 0.018 for AIDR 3D). Analysis of the anatomical phantoms thus neither confirmed the superiority of AIDR 3D nor dose effects observed in the uniform phantom.

**Table 4:** Detection accuracy (%) per dose and reconstruction method in anatomical phantom images. Averaged results across all lesion contrasts and 95% confidence intervals are presented

	FBP	AIDR 3D	*p* value
1.4 mGy	68.2 (56.9 to 79.5)	73 (62.3 to 83.7)	*0.144*
5.6 mGy	74.6 (64.2 to 85.1)	81.1 (72.8 to 89.3)	*0.111*
*p* value	*0.027*	*0.018*	

### Noise characteristics

**Fig. 6:**
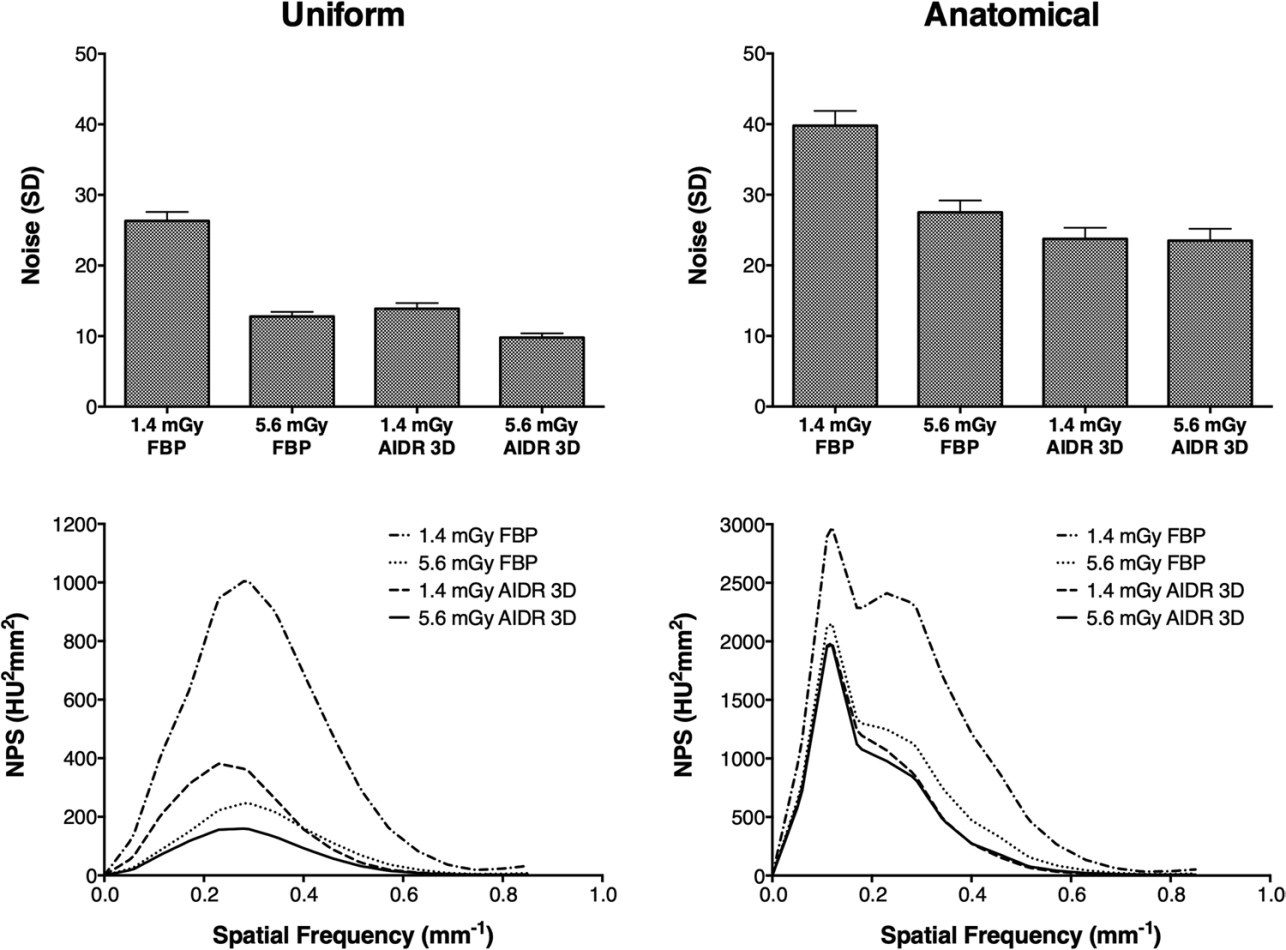
Noise and noise power spectrum (NPS) results. Average noise values from 200 images per phantom type, dose, and image reconstruction are presented. Error bars indicate standard deviations

Figure 6 shows noise and NPS results per phantom type, dose, and image reconstruction. Numerical results are summarized in Table 5. As expected, low-dose (1.4 mGy), FBP-reconstructed images had the highest noise level in both phantom types (*p* < 0.001). A dose increase to 5.6 mGy reduced the noise (*p* < 0.001) except for AIDR 3D-reconstructed images of the anatomical phantoms, which had almost identical noise values at low and high doses (*p* = 0.26). Remarkably, noise was lower in low-dose AIDR 3D-reconstructed images than in high-dose FBP-reconstructed images of the anatomical, but not of the uniform, phantom, indicating that AIDR 3D was more effective in denoising anatomical images. The NPS curves of the uniform phantom showed a shift towards lower spatial frequencies in low-dose AIDR 3D-reconstructed images with a peak NPS at 0.23 mm^−1^ and a decrease at lower spatial frequencies. Conversely, all images of the anatomical phantoms yielded peak NPS values at a low spatial frequency of 0.12 mm^−1^ regardless of dose and image reconstruction. FBP-reconstructed images acquired at 1.4 mGy had a second NPS peak at a spatial frequency of 0.23 mm^−1^, which flattened with FBP reconstruction at 5.6 mGy and in all images reconstructed with AIDR 3D.

**Table 5:** Noise (SD) and spatial frequency (mm^−1^) of the peak amplitude of the noise power spectrum (NPS). Averaged noise results from 200 images per phantom type, dose, and image reconstruction are presented along with 95% confidence intervals

	1.4 mGy FBP	1.4 mGy AIDR 3D	5.6 mGy FBP	5.6 mGy AIDR 3D
Noise (SD) Uniform	26.33 (26.15 to 26.51)	13.89 (13.77 to 14)	12.79 (12.7 to 12.88)	9.8 (9.72 to 9.88)
Noise (SD) Anatomical	39.79 (39.5 to 40.08)	23.76 (23.54 to 23.98)	27.52 (27.28 to 27.75)	23.5 (23.27 to 23.74)
Spatial frequency of peak NPS (mm^−1^) Uniform	0.28	0.23	0.29	0.28
Spatial frequency of peak NPS (mm^−1^) Anatomical	0.12	0.12	0.12	0.12

## Discussion

Task-based methods have been proposed to evaluate and compare CT techniques for their diagnostic performance in clinical practice. Task-based assessment is typically performed using CT images of uniform phantoms, and it is of interest to what extent evidence from uniform phantoms actually reflects detectability in clinical images with anatomical detail. The present study therefore compared low-contrast detectability between uniform and anatomically realistic phantoms. Our results show that anatomical phantom structure affects detection accuracy at all investigated lesion contrasts (*p* < 0.001), interferes with dose effects on detection and influences the assessment of AIDR 3D performance compared to FBP.

The image assessment results we obtained for the uniform phantom are in good agreement with previous reports of relatively high detection sensitivities of more than 87% for lesions of the same size as investigated in our study [13, 14]. Anatomical phantom structure significantly impaired lesion detectability—a contrast increase to 30 HU was necessary to achieve similar detection accuracy as for 9 HU lesion contrast in uniform images. Near-perfect detectability was achieved at a markedly higher lesion contrast (38 HU) than with the uniform phantom (18 HU).

An impact of anatomical detail was expected because structured tissue patterns (anatomical noise) have psychophysical effects on humans that interfere with detection tasks. Previous X-ray studies found anatomical noise to have stronger effects than quantum noise and to impair and eventually limit human lesion perception [5–7]. This, in turn, may influence how dose changes affect detection tasks [6, 7]. Our experiments confirm the effects of anatomical patterns on noise characteristics and the assessment of dose and reconstruction methods. Anatomical images had a low-frequency noise component that was predominant regardless of dose and image reconstruction mode. This component was in good agreement with reports of high NPS values at low spatial frequencies in patients [15]. Anatomical background structure also influenced the denoising power of AIDR 3D, which adds to reports on interactions between anatomical texture, noise, and spatial resolution when iterative reconstruction is applied [16–18]. Lesion detectability was clearly affected by dose in uniform FBP images. However, the dose-detection relationship was less clear in images with anatomical noise. Consistent with published results, AIDR 3D maintained detectability and was superior to FBP at a lower dose in uniform phantom images [19]. These advantages were lost when anatomical structures interfered with lesion detection.

Significant texture effects on detectability were also observed in a previous CT study that compared liver-mimicking textures with a uniform phantom background [8]. In that study, structured background textures reduced the influence of dose changes on detection, similar to what we observed for FBP images. Another CT study came to different conclusions and reported only negligible effects of liver texture on detectability in comparison with a water background [9]. However, liver and water textures in that study were visually quite similar, which explains why the results differ from our observations. However, it should also be noted that the comparability of our results with both of these CT studies is limited by differences in CT hardware and because both studies investigated cropped images with vessel-free liver textures. To the best of our knowledge, our study is the first to compare neck phantom images with full anatomical detail, which is relevant because anatomical detail adds complexity to CT images and has a relevant impact on human lesion perception [20, 21].

The experiments we performed here do not provide an in-depth analysis of dose reduction and image reconstruction, which requires broader testing and can be found elsewhere [22]. For example, AIDR 3D was reported to have similar performance as FBP at 120 kVp, which our experiments confirmed, and also to be superior at a lower tube voltage of 100 kVp, which we did not assess [22]. Our study evaluated the effects of phantom background on task-based CT assessment, and we used two dose levels and reconstructions methods to illustrate such effects. Based on our results, we conclude that phantom background has a relevant influence and that transferability of CT assessment to clinical imaging can be expected to improve as the realism of the test environment increases. In view of the published evidence discussed above, we believe that this should apply beyond the CT scanner and imaging technologies used here.

The limitations of our study include the rather narrow study protocol, which was selected to investigate the effects of phantom background, but not to perform a comprehensive analysis of dose and image reconstruction methods. Results may differ in less complex anatomical regions than the neck. However, the generalizability of our results is supported by previous work in liver imaging, which has arrived at similar conclusions about the importance of phantom texture [8]. It should also be noted that we deliberately chose a location-known-exactly experimental design in order to avoid introducing different lesion locations as another variable possibly influencing detectability. Yet, detection experiments with lesions in unknown locations can be considered to be more realistic and representative of clinical image interpretation [4].

Uniform phantoms differ from patients and provide an idealized environment for evaluating CT systems. Our results provide evidence that lesion contrasts in CT images of uniform phantoms are below those that are clinically relevant and corroborate data indicating that anatomical phantom structure affects estimates of CT performance and reasonable dose selection. Investigations of CT assessment aimed at predicting and comparing clinical performance must take into account differences between phantoms and patients and should be performed in a setting that mimics clinical imaging as closely as possible.

## Supplementary Information

Below is the link to the electronic supplementary material.

Supplementary file1 (DOCX 48 KB)

## Abbreviations

4-AFC: 4-Alternative forced choice
AIDR 3D: Adaptive iterative dose reduction 3D
CT: Computed tomography
CTDI: Computed tomography dose index
FBP: Filtered back projection
HU: Hounsfield unit

## Declarations

### Guarantor

The scientific guarantor of this publication is Dr. Paul Jahnke.

### Statistics and biometry

No complex statistical methods were necessary for this paper.

### Study subjects or cohorts overlap

Some study subjects or cohorts have been previously reported in Conzelmann J, Schwarz FB, Hamm B, Scheel M, Jahnke P (2020) Development of a method to create uniform phantoms for task-based assessment of CT image quality. J Appl Clin Med Phys 21:201–208.

### Methodology

• prospective

• observational

• performed at one institution
