# Supplementary material for: Comparison of low-contrast detectability between uniform and anatomically realistic phantoms—influences on CT image quality assessment
Source: Eur Radiol. 2021 Sep 2;32(2):1267–75. doi: 10.1007/s00330-021-08248-3 (PMC8794946; doi:10.1007/s00330-021-08248-3)
Supplement: Supplementary file 1 — Supplementary file1 (DOCX 48 KB) [file 330_2021_8248_MOESM1_ESM.docx]

**Suppl. table**

Suppl. table 1: Confidence scores per phantom type and lesion contrast. Averaged results across all readers, dose levels, and reconstruction methods at 4, 9 (uniform), 10 (anatomical), 18, 30, and 38 HU lesion contrast are presented along with 95% confidence intervals.

|  | **4 HU lesion contrast** | **9/10 HU lesion contrast** | **18 HU lesion contrast** | **30 HU lesion contrast** | **38 HU lesion contrast** |
| --- | --- | --- | --- | --- | --- |
| **Uniform** | 1.58  (1.31 to 1.84) | 2.68  (2.22 to 3.14) | 4.11  (3.73 to 4.5) | 4.73  (4.54 to 4.91) | 4.94  (4.88 to 5) |
| **Anatomical** |  | 1.33  (1.12 to 1.54) | 1.33  (1.13 to 1.52) | 2.15  (1.81 to 2.48) | 3.15  (2.78 to 3.52) |
| ***p-value*** |  | *< 0.001* | *< 0.001* | *< 0.001* | *< 0.001* |
